# Bronchial artery chemoembolization in the treatment of refractory central lung cancer with atelectasis

**DOI:** 10.3389/fonc.2024.1343324

**Published:** 2024-06-12

**Authors:** Yujin Liu, Xiumei Zhang, Fenxiang Zhang, Weixiang Song

**Affiliations:** ^1^ Department of Interventional Oncology, Yueyang Hospital of Integrated Traditional Chinese and Western Medicine, Shanghai University of Traditional Chinese Medicine, Shanghai, China; ^2^ Nursing department, Tongji University Affiliated Shanghai Fourth People’s Hospital, Shanghai, China

**Keywords:** bronchial artery chemoembolization, lung cancer, atelectasis, treatment effects, case series

## Abstract

**Objective:**

This study aims to explore the clinical application of bronchial artery chemoembolization (BACE) in managing refractory central lung cancer with atelectasis.

**Methods:**

The retrospective case series includes patients diagnosed with refractory central lung cancer and atelectasis who underwent BACE treatment at Yueyang Integrated Traditional Chinese and Western Medicine Hospital, affiliated with Shanghai University of Traditional Chinese Medicine, from January 2012 to December 2021.

**Results:**

All 30 patients with lung cancer successfully underwent BACE procedures. Their ages ranged from 62 to 88 years, with an average age of 67.53. The treatment interval was 21 days, and the treatment cycle ranged from 2 to 12 times, averaging 4.13 times. During the BACE procedures, the Karnofsky Performance Status (KPS) score after 2 to 3 BACE cycles showed a significant improvement (82.0 ± 10.1 vs 68.3 ± 14.0, P < 0.001) than that of before BACE. Only nutritional support and symptomatic treatment were performed after BACE, and no major hemoptysis were observed. During follow-up, 23 cases resulted in mortality, while seven survived. The median progression-free survival (PFS) and overall survival (OS) were 7.0 (95% CI: 4.6–9.4) and 10.0 (95% CI: 6.2–13.8) months, respectively, with 1-, 2-, and 3-year survival rates of 84.0%, 53.5%, and 11.3%, respectively. Eight cases exhibited bronchial recanalization and relief of atelectasis. According to the RECIST scale, there were 4 cases of complete response (CR), 16 cases of partial response (PR), 9 cases of stable disease (SD), and 1 case of progressive disease (PD). No serious adverse events were reported.

**Conclusion:**

BACE might be a safe intervention for refractory central lung cancer accompanied by atelectasis. The procedure exhibits satisfactory outcomes in tumor control, atelectasis relief, and enhancement of quality of life, warranting further investigation.

## Highlights

This study investigates the clinical application of bronchial artery chemoembolization (BACE) in managing refractory central lung cancer with atelectasis. The retrospective case series, conducted at Yueyang Integrated Traditional Chinese and Western Medicine Hospital, includes 30 patients diagnosed with this condition between January 2012 and December 2021. The BACE procedures were successfully performed on all patients, leading to significant short-term symptom improvements and Karnofsky performance status (KPS) scores. The median progression-free survival (PFS) and overall survival (OS) were 7 months and 10 months, respectively, with encouraging 1-, 2-, and 3-year survival rates.

The findings suggest that BACE is a safe intervention, offering favorable outcomes in tumor control, atelectasis relief, and quality of life enhancement. The observed increase in KPS scores indicates an improvement in overall patient well-being. Importantly, no serious adverse events were reported. Given these promising results, further investigation is warranted. The manuscript is well-suited for submission to a medical journal specializing in interventional oncology, respiratory medicine, or integrative medicine. The study’s focus on a novel intervention for refractory central lung cancer addresses a critical clinical challenge. It contributes valuable insights to the field, making it a strong fit for journals interested in innovative approaches to cancer management.

## Introduction

Central lung cancer denotes lung cancer that originates in the trachea, bronchial tree, major blood vessels, esophagus, or heart (1). Central lung cancers are usually ineligible for surgery, and the treatment options include radiotherapy and systemic therapies (chemotherapy, targeted therapy, and immunotherapy) (2–6). Central lung cancer is frequently accompanied by atelectasis (1). Atelectasis primarily arises from tumor obstruction and compression by metastatic lymph nodes, often coexisting with obstructive pneumonia (7). Patients facing malignant central airway obstruction not only contend with an unfavorable overall prognosis but also endure severe respiratory symptoms and a diminished quality of life (8–10). The management of the case under discussion was particularly arduous due to the disease’s advanced stage and the patient’s deteriorating clinical state, marked by recurrent pulmonary infections and severe respiratory distress stemming from central airway obstruction (11, 12). While bronchoscopy and stent placement represent the primary approaches for addressing atelectasis, they do not constitute a cure for the underlying tumor (13–15).

Ever since Viamonte’s pioneering bronchial artery angiography in 1964, a growing array of local intervention treatments, including bronchial artery embolization and chemoembolization, have emerged, instilling hope for the survival of lung cancer patients (16–18). In recent years, interventional therapy technologies have been widely applied in lung cancer treatment, offering advantages such as minimally invasive procedures, minimal adverse reactions, precise therapeutic outcomes, and repeatability. These advancements have significantly enhanced the survival rates and quality of life for individuals with lung cancer (19–21).

However, existing research focuses predominantly on bronchial arterial chemoembolization (BACE) and lung cancer centers on non-small cell lung cancer (NSCLC). For instance, in 2023, Yu et al. compared the efficacy and safety of drug-eluting bead BACE against conventional BACE in NSCLC (22). Similarly, in 2021, Liu et al. investigated the effectiveness and safety of drug-eluting bead BACE in combination with oral anlotinib for treating NSCLC (23). The application of BACE in addressing central lung cancer with atelectasis remains notably underreported. Recognizing the inherent challenges in treating central lung cancer, our center has, over the past decade, employed transcatheter BACE for central lung cancer complicated by atelectasis, yielding discernible therapeutic outcomes. Thus, this study aims to explore the clinical application of BACE in managing refractory central lung cancer with atelectasis.

## Methods

### Study design and patients

We conducted a retrospective analysis of patients diagnosed with locally advanced (IIIB, IIIC) and advanced (IVA, IVB) refractory central lung cancer complicated with atelectasis who underwent BACE treatment at Yueyang Integrated Traditional Chinese and Western Medicine Hospital affiliated with Shanghai University of Traditional Chinese Medicine from January 2012 to December 2021. The inclusion criteria were: 1) Histopathological confirmation of lung cancer; 2) Completion of at least one BACE treatment; 3) Availability of complete follow-up information. The study was approved by the ethics committee of Yueyang Integrated Traditional Chinese and Western Medicine Hospital affiliated with the Shanghai University of Traditional Chinese Medicine. The approval number is 2021–008.

### Treatment

Patients underwent successful BACE procedures conducted under local anesthesia. Using the modified Seldinger technique, the right or left femoral artery was punctured, followed by transaortic selective arterial cannulation of the left or right bronchial arteries. Two micocatheter models were used. One was Stride Micocatheter (Asahi STD125–26S) with an outer diameter of 2.6Fr (0.88mm) at the head end and a Micro Guidewire with an outer diameter of 0.018Fr (0.46mm). Straight head guide wire, can be molded into a J shape. Another Merit Maestro Micocatheter (Merit Medical Syetem, 29MC29130ST), head end outer diameter is 2.9Fr (0.96mm), Swan neck shape, Inside Steerable Guidewire (Merit Medical Tenor, TNR2811), outer diameter 0.018Fr (0.46mm), straight head guide wire, can be molded into J shape according to demand. Digital subtraction angiography (DSA) confirmed the bronchial artery’s blood supply to the target tumor and ruled out abnormal shunting to the spinal cord and brain. Paclitaxel (60–80 mg/m^2^) and cisplatin (30–40 mg/m^2^) or carboplatin (dose calculated as AUC=5) were diluted to 50ml-100ml with 0.9% sodium chloride solution and slowly infused into the tumor area through the bronchial artery, taking at least 20 minutes. Subsequently, the bronchial artery endings were embolized using 150–350-560 µm gelatin sponge particles. Following the procedure, the catheter was removed, the puncture site was pressed for hemostasis and bandaged, and the patient remained in bed for 8 hours.

### Data collection and definition

The efficacy evaluation was grounded in patient responsiveness and disease status, employing the Response Evaluation Criteria in Solid Tumors (RECIST) scale (24). This scale categorizes outcomes as follows: complete response (CR), denoting the disappearance of all target lesions, with any pathological lymph nodes showing a reduction in short axis to <10 mm; partial response (PR), indicating at least a 30% decrease in the sum of diameters of target lesions, using the baseline sum diameters as a reference; stable disease (SD), characterized by neither sufficient shrinkage for PR nor sufficient increase for progressive disease (PD), with the smallest sum diameters taken as reference during the study; and PD, involving at least a 20% increase in the sum of diameters of target lesions, using the smallest sum on study as reference (including the baseline sum if that is the smallest on study). Moreover, the sum must demonstrate an absolute increase of at least 5 mm, and the appearance of one or more new lesions is also considered progression. Patient outcomes were used to determine progression-free survival (PFS), overall survival (OS), Karnofsky performance status (KPS) scores, and adverse events. PFS and OS were calculated from the first BACE procedure.

### Statistical analysis

All statistical analyses were performed using SPSS 25.0 for Windows (IBM, Armonk, NY, USA). Continuous and categorical variables were presented as mean ± standard deviation and n (%), respectively. PFS and OS were assessed using the Kaplan-Meier method and log-rank test. Two-sided P-values <0.05 were considered statistically significant.

## Results

### Characteristics of the patients

The clinical characteristics of all 30 lung cancer patients are summarized in **Table 1**. Their ages ranged from 62 to 88 years, with an average age of 68. PD-L1 expression was <1% in all included patients, and no immunotherapy was performed. Seventeen patients (56.7%) underwent radiotherapy, chemotherapy, and molecular-targeted therapy before BACE.

**Table 1:** Clinical characteristics of patients.

Characteristic	N=30
Age, mean	67.53 ± 9.662
Gender	
Male Female	29 (96.7) 1 (3.3)
KPS	
Before BACE After BACE	68.30 ± 14.01 82.00 ± 10.07
ECOG	
Before BACE After BACE	1.93 1.13
Pathological type	
Squamous cell carcinoma of the lung	18 (60.0)
Lung adenocarcinoma	10 (33.3)
Small cell carcinoma of the lung	2 (6.7)
T stage	
T3 T4	11 (36.7) 19 (63.3)
N stage	
N1 N2 N3	1 (3.3) 8 (26.7) 21 (70.0)
Clinical stages	
IIIB IIIC	10 (33.3) 2 (6.7)
IVA IVB	7 (23.3) 11 (36.7)
Treatment (chemotherapy, radiotherapy, targeted)	17 (56.7)

KPS, Karnofsky performance status; BACE, bronchial artery chemoembolization; PS, performance status; ECOG, Eastern Cooperative Oncology Group.

### Characteristics of the BACE procedures

The BACE procedure was successfully performed on all subjects. The treatment interval was 21 days, and the treatment cycle ranged from 2 to 12 times, averaging 4.1 cycles. The Karnofsky Performance Status (KPS) score showed a significant improvement from 68.3 ± 14.0 before BACE to 82.0 ± 10.1 after 2 to 3 BACE cycles (P < 0.001). Only nutritional support and symptomatic treatment were performed after BACE. There were no cases of major hemoptysis. Since the cases of hemoptysis were mild, the procedure was the same.

### Follow-up and survival

**Figure 1:**
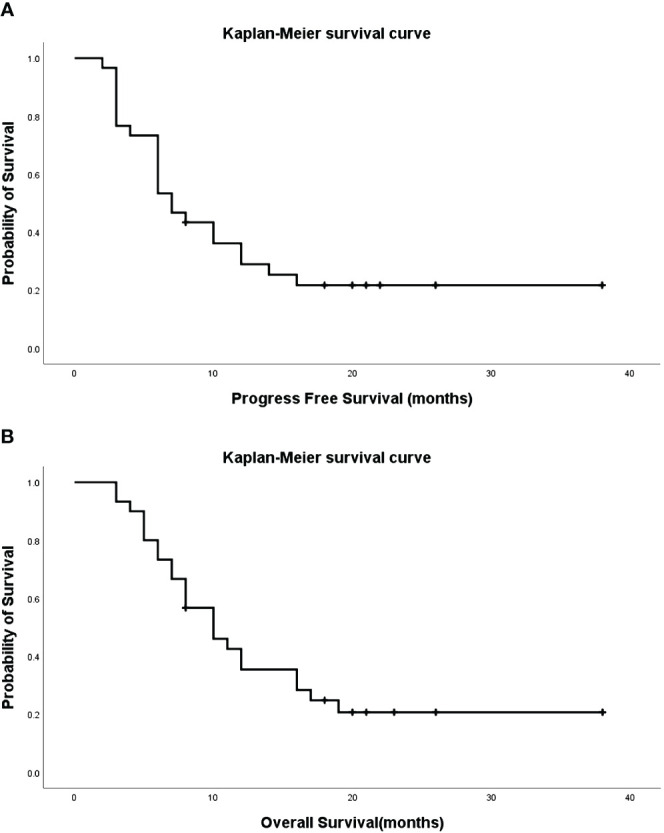
Progression-Free Survival (PFS) [**(A)**, median=7.0, 95%CI: 4.6–9.4] and Overall Survival (OS) [**(B)**, median=10.0, 95%CI: 6.2–13.8] of patients.

During follow-up, 23 cases resulted in mortality, while seven survived. The median progression-free survival (PFS) and overall survival (OS) were 7.0 (95% CI: 4.6–9.4) and 10.0 (95% CI: 6.2–13.8) months, respectively (**Figure 1**), with 1-, 2-, and 3-year survival rates of 84.0%, 53.5%, and 11.3%, respectively. Eight cases exhibited bronchial recanalization and relief of atelectasis. According to the RECIST scale, there were 4 cases of complete response (CR), 16 cases of partial response (PR), 9 cases of stable disease (SD), and 1 case of progressive disease (PD). No serious adverse events related to the procedure were reported. Patients with progressive disease were transitioned to radiotherapy, best supportive treatment, or hospice care. One patient died due to an accident, who had complications, including cough, respiratory failure, heart failure, and dysphoria.

### Presentation of typical cases

**Figure 2:**
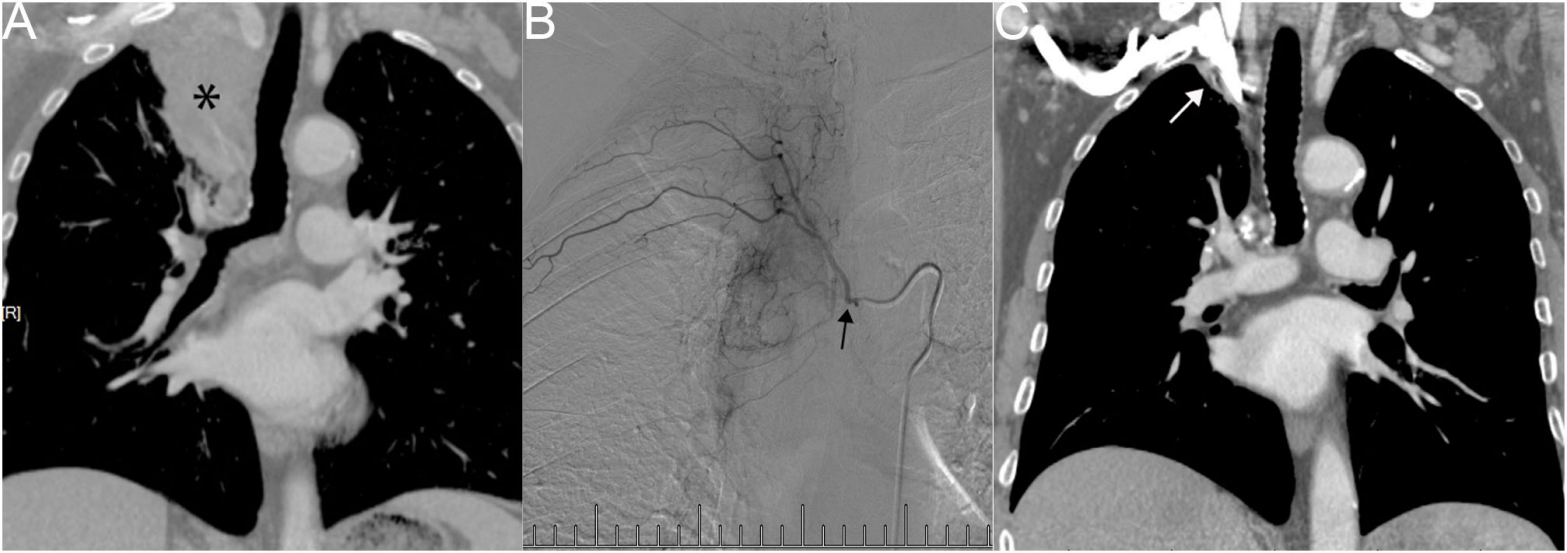
Male, 83 years old, with a right upper lung tumor, discovered due to chest pain, confirmed by biopsy as squamous cell carcinoma (T4N3M0). **(A)** CT Coronal plane image showing right upper lung tumor with atelectasis of the right upper lobe, hilar, and mediastinal lymph node metastasis (*). **(B)** A right bronchial artery angiography indicating blood supply to the right lung tumor, which can be perfused with chemotherapy and embolization (→). **(C)** CT Coronal plane image after six courses of BACE, demonstrating that the right upper lung tumor and atelectasis have basically disappeared (→).

One patient, diagnosed by biopsy with squamous cell carcinoma (T4N3M0), exhibited a right upper lung tumor with atelectasis, hilar, and mediastinal lymph node metastasis (**Figure 2A**). In **Figure 2B**, right bronchial artery angiography revealed blood supply to the right lung tumor, amenable to chemotherapy and embolization. After six courses of BACE, the CT coronal plane image displayed the disappearance of the right upper lung tumor and atelectasis (**Figure 2C**).

**Figure 3:**
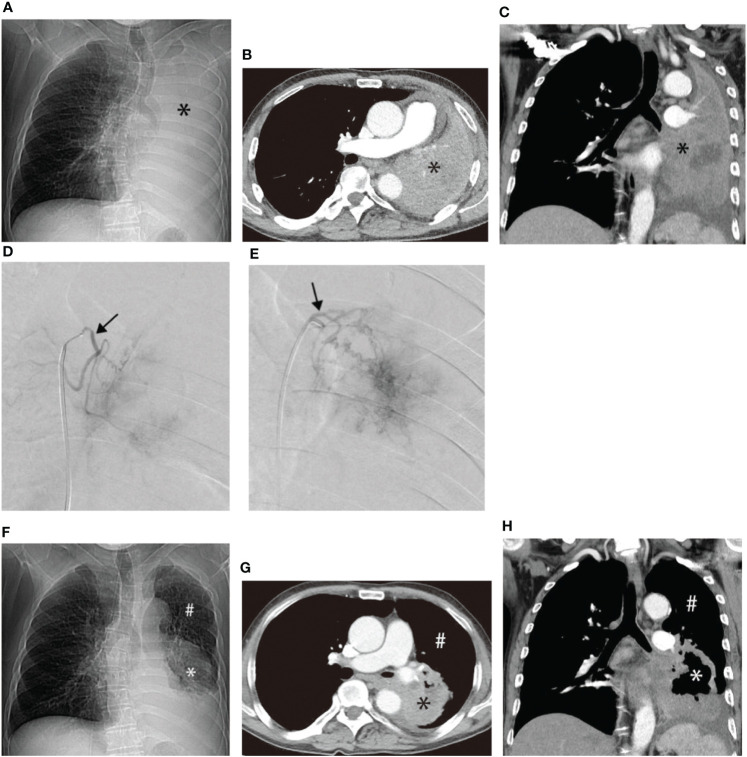
Male, 62 years old, experiencing progression of left lung squamous cell carcinoma after systemic chemotherapy, resulting in chest tightness and difficulty breathing after activity. **(A–C)** display chest plain film, CT cross-section, and CT Coronal plane, respectively, showing complete consolidation of the left lung (*). **(D, E)** illustrate the blood supply to the tumor through two bronchial arteriography images, respectively (→). **(F–H)** represent chest plain film, CT cross-section, and CT Coronal plane after two BACE courses, respectively, showing left lung recruitment (#), left hilar tumor necrosis, and cavity formation (*).

Another patient with left lung squamous cell carcinoma, experiencing chest tightness and difficulty breathing post-systemic chemotherapy, demonstrated complete consolidation of the left lung on chest plain film, CT cross-section, and CT coronal plane (**Figures 3A–C**). Two bronchial arteriography images revealed the tumor’s blood supply (**Figures 3D, E**). After two BACE courses, the left lung was recruited, left hilar tumor necrosis occurred, and a cavity formed (**Figures 3F–H**).

**Figure 4:**
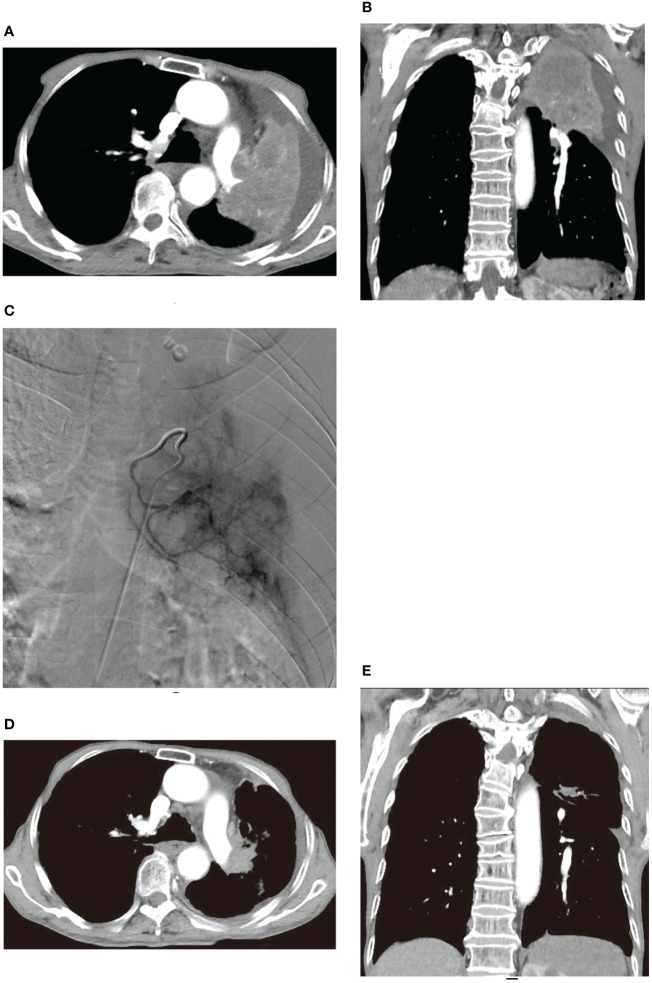
An 84-year-old male developed cough, bloody sputum, and chest tightness after chemotherapy for left lung squamous cell carcinoma. **(A, B)** are CT transverse and Coronal plane images, respectively, showing left upper lung consolidation with a small amount of effusion. **(C)** displays a left bronchial artery angiography, revealing a tumor supplying the left lung with abundant blood supply to the cancer. **(D, E)** are CT transverse and Coronal plane images, respectively, illustrating that the left upper lung is dilated, with a small amount of residual lesions and effusion disappeared.

**Figure 5:**
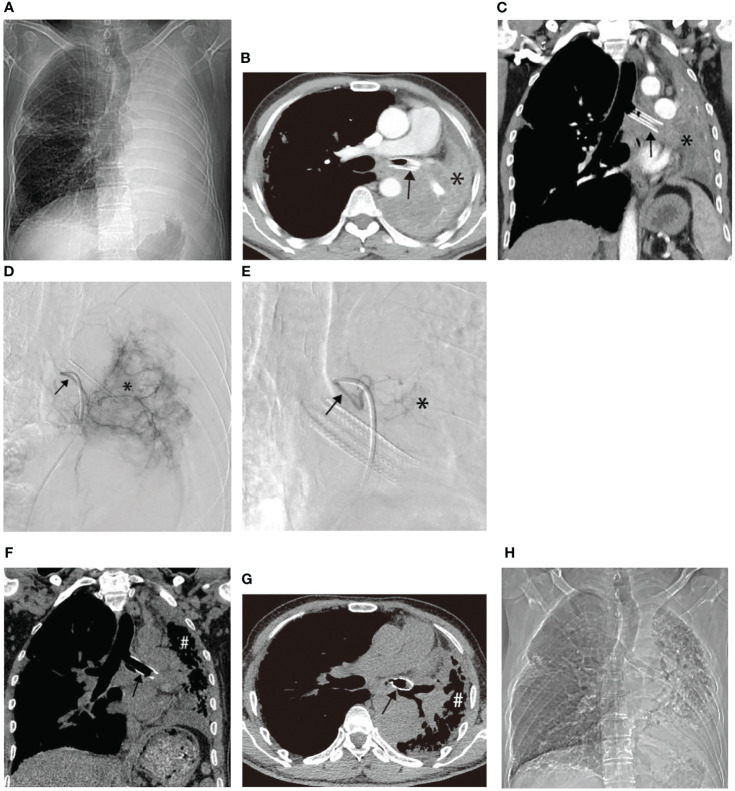
A 58-year-old male with left lung squamous cell carcinoma experienced atelectasis and difficulty breathing after radiotherapy, chemotherapy, and placement of a left main bronchial stent. **(A–C)** show chest plain film, CT cross-section, and CT coronal plane, respectively, displaying complete consolidation of the left lung (*) and the left main bronchial stent (→). **(D, E)** illustrate the blood supply to the tumor through two bronchial arteries, respectively (→). **(F–H)** exhibit chest plain films, CT cross-section images, and CT coronal images after one BACE, respectively, revealing partial dilation of the left lung (#) and relief of dyspnea in the patient.

Similar improvements were observed in two other patients before and after treatment, as illustrated in **Figures 4**, **5**. **Figure 4** showcases an 84-year-old man with left lung squamous cell carcinoma who developed cough, phlegm, and chest tightness after chemotherapy. **Figures 4A, B** depicts left upper lung consolidation with minor effusion, while 4C reveals left bronchial artery angiography supplying the tumor. In **Figures 4D, E**, the left upper lung dilated with a small residual lesion, and effusion disappeared. **Figure 5** displays a 58-year-old man with left lung squamous cell carcinoma experiencing atelectasis and breathing difficulty after radiotherapy, chemotherapy, and the placement of a left main bronchial stent. **Figures 5A–C** shows complete consolidation of the left lung and left main bronchial stent. **Figures 5D, E** reveals the tumor’s blood supply through two bronchial arteries. **Figures 5F–H** shows a partially dilated left lung and reduced dyspnea.

### BACE-related complications

There were some complications related to BACE treatment (**Table 2**). Most of them were grade 1–2. Grade 1–2 cough and chest pain after BACE usually resolved within a few hours. The hematopoietic system was impaired in some patients. Fatigue, hair loss, and constipation were common, but mostly 1–2 grades. Occasionally, there was subcutaneous ecchymosis at the femoral artery puncture point, which was probably a failure of hemostasis due to pressure.

**Table 2:** Complications of BACE, according to the WHO standards (25).

Complication	Grade 1	Grade 2	Grade 3
Cough	2	3	
Thoracodynia	5	2	
Leukocytosis	2		
Thrombocytopenia			1
Fatigue	13	2	
alopecia	6	18	
Constipation	8	6	3
Puncture bleeding	2		

## Discussion

Central lung cancers are often ineligible for surgery, and the treatment options are limited to radiotherapy and systemic therapies (chemotherapy, targeted therapy, and immunotherapy) (2–6). Still, central lung cancer carries a poor prognosis (26), and exploring novel approaches to improve prognosis is important. In this study, we conducted a retrospective analysis of 30 cases of refractory central lung cancer complicated with atelectasis treated by BACE at our center over the past decade. The results demonstrated that the short-term curative effect of BACE significantly alleviated symptoms such as cough, sputum, hemoptysis, dyspnea, and hypoxia, improved the KPS score, and showed no serious adverse events. BACE-related toxic side effects were mild, and the patients easily accepted BACE, especially those with poor physical status. These findings offer valuable insights into diagnosing and treating central lung cancer.

As acknowledged, angiogenesis plays a crucial role in tumor growth and serves as the anatomical foundation for regional therapy via blood vessels (27, 28). The primary blood supply arteries for lung cancer involve systemic circulation arteries, including the bronchial artery, intercostal artery, and phrenic artery (29, 30). BACE infusion chemotherapy through the bronchial artery maintains a high local drug concentration in tumor tissue, enhancing the effective eradication of tumor cells. This localized approach minimizes drug concentration in vital organs, consequently reducing systemic adverse reactions (31, 32), aligning with our study results. Following BACE treatment in our study, there was a significant short-term efficacy, improvement in KPS score, and no serious adverse events. Furthermore, bronchial recanalization and atelectasis were relieved in 8 patients, with enhanced lung function observed post-treatment. This improvement could be attributed to the gradual shrinkage or necrosis of the tumor after interventional treatment, alleviating dyspnea and venous return obstruction caused by tumor invasion and bronchial compression, ultimately enhancing the lung function of patients (33).

Additionally, bronchial artery embolization, by blocking local blood flow, prolongs the residence time of chemotherapy drugs in tumor tissue, thereby exerting a more potent anti-tumor effect (34). Wang et al. observed that BACE for lung cancer exhibited fewer adverse reactions, a shorter treatment course, and superior curative effects compared to systemic intravenous chemotherapy. For patients ineligible or unwilling to undergo surgery, it effectively improves the quality of life, extends survival periods, and, in some cases, offers the opportunity for secondary surgical eradication (35), representing a clinically valuable and comprehensive therapy. Moreover, Shang et al. reported an objective response rate of 69.45% and 58.33% at three and six months, respectively, after DEB-BACE in advanced non-small cell lung cancer, with disease control rates of 88.89% and 83.33% (36). While these studies confirm the value of BACE in lung cancer, our study is the first to explore the efficacy of BACE in central lung cancer with atelectasis.

Self-expandable metallic stents can be used for malignant obstructive atelectasis (37), but their use is relatively recent, and they have only recently been approved for that use in China. Some patients received stents in the present study but no self-expandable metallic stents. In addition, embolization microspheres are available, but the authors used gelatine sponge granule embolization for BACE mainly for patient safety. Recently, the use of drug-eluting beads (DEBs) has been reported. Shang et al. (36) reported an ORR of 69.45% and 58.33% at 3 and 6 months, respectively, after DEB-BACE in patients with advanced non-small cell lung cancer, with DCRs of 88.89% and 83.33%. On the other hand, serious side effects were also reported, including massive hemoptysis and death by asphyxia due to hemoptysis (38). Therefore, it is the authors’ opinion that such materials should be used with caution and only in selected patients.

The study has several limitations that need to be acknowledged. Firstly, due to its retrospective and observational nature, the study is inherently susceptible to retrospective bias and cannot establish causation (39). Secondly, the study’s small sample size and the fact that all participants came from a single center could introduce bias into the outcomes. Therefore, it is important to approach the findings of this study with caution. Thirdly, two patients with small cell lung cancer were included; this study did not focus on any specific type of lung cancer, only on refractory central lung cancer complicated with atelectasis and treated using BACE, and BACE was effective in these two cases. Although no conclusion can be drawn about the type of lung cancer, those two cases were kept. Fourthly, many patients were initially diagnosed and treated at other hospitals and referred only for BACE. Some data were missing in such cases and could not be analyzed in the present study. Consequently, validating these results requires a large-scale, randomized study conducted across multiple centers.

In conclusion, it is anticipated that the role of BACE in lung cancer treatment will gain increasing recognition and refinement. However, the value of BACE in treating central lung cancer with atelectasis requires further substantiation.

## Data availability statement

The original contributions presented in the study are included in the article/supplementary material. Further inquiries can be directed to the corresponding author.

## Ethics statement

The study received approval from the ethics committee of Yueyang Integrated Traditional Chinese and Western Medicine Hospital affiliated with Shanghai University of Traditional Chinese Medicine. The approval number is 2021-008. The studies were conducted in accordance with the local legislation and institutional requirements. The ethics committee/institutional review board waived the requirement of written informed consent for participation from the participants or the participants’ legal guardians/next of kin because this study is retrospective and does not require informed consent.

## Author contributions

YL: Conceptualization, Methodology, Project administration, Resources, Writing – original draft, Writing – review & editing. XZ: Conceptualization, Formal analysis, Methodology, Resources, Writing – original draft, Writing – review & editing. FZ: Formal analysis, Investigation, Methodology, Resources, Writing – original draft, Writing – review & editing. WS: Project administration, Resources, Software, Supervision, Writing – original draft, Writing – review & editing.
